# Osteocytes and the pathogenesis of hypophosphatemic rickets

**DOI:** 10.3389/fendo.2022.1005189

**Published:** 2022-09-29

**Authors:** Miwa Yamazaki, Toshimi Michigami

**Affiliations:** Department of Bone and Mineral Research, Research Institute, Osaka Women’s and Children’s Hospital, Osaka Prefectural Hospital Organization, Izumi, Japan

**Keywords:** phosphate, osteocyte, fibroblast growth factor 23, rickets, mutation

## Abstract

Since phosphorus is a component of hydroxyapatite, its prolonged deprivation affects bone mineralization. Fibroblast growth factor 23 (FGF23) is essential for maintaining phosphate homeostasis and is mainly produced by osteocytes. FGF23 increases the excretion of inorganic phosphate (Pi) and decreases the production of 1,25-dihydroxyvitamin D in the kidneys. Osteocytes are cells of osteoblastic lineage that have undergone terminal differentiation and become embedded in mineralized bone matrix. Osteocytes express *FGF23* and other multiple genes responsible for hereditary hypophosphatemic rickets, which include *phosphate-regulating gene homologous to endopeptidase on X chromosome* (*PHEX*), *dentin matrix protein 1* (*DMP1*), and *family with sequence similarity 20, member C* (*FAM20C*). Since inactivating mutations in *PHEX*, *DMP1*, and *FAM20C* boost the production of FGF23, these molecules might be considered as local negative regulators of FGF23. Mouse studies have suggested that enhanced FGF receptor (FGFR) signaling is involved in the overproduction of FGF23 in *PHEX*-deficient X-linked hypophosphatemic rickets (XLH) and *DMP1*-deficient autosomal recessive hypophosphatemic rickets type 1. Since FGFR is involved in the transduction of signals evoked by extracellular Pi, Pi sensing in osteocytes may be abnormal in these diseases. Serum levels of sclerostin, an inhibitor Wnt/β-catenin signaling secreted by osteocytes, are increased in XLH patients, and mouse studies have suggested the potential of inhibiting sclerostin as a new therapeutic option for the disease. The elucidation of complex abnormalities in the osteocytes of FGF23-related hypophosphatemic diseases will provide a more detailed understanding of their pathogenesis and more effective treatments.

## Introduction

In human adults, approximately 90% of total phosphorus in the body is stored in the skeleton as calcium/phosphate crystals, such as hydroxyapatite. Most of the remaining phosphorus exists in soft tissues, and less than 1% is distributed in extracellular fluid (ECF). Phosphorus is present as inorganic phosphate (Pi) in serum (1). Since Pi is indispensable for the synthesis of hydroxyapatite, its prolonged deficiency or wasting impairs skeletal mineralization (2).

Fibroblast growth factor 23 (FGF23) is produced in bone and increases renal Pi excretion by down-regulating the expression of type IIa and IIc sodium/Pi (Na^+^/Pi) co-transporters (NaPi-IIa and NaPi-IIc, respectively) (3). FGF23 also down-regulates the expression of 25-hydroxyvitamin D-1α hydroxylase and up-regulates that of 25-hydroxyvitamin D-24 hydroxylase, reducing the production of 1,25-dihydroxyvitamin D (1,25(OH)_2_D) (3). The excessive effects of FGF23 result in hypophosphatemic rickets/osteomalacia, which is characterized by renal Pi wasting, hypophosphatemia, inappropriately normal or low levels of serum 1,25(OH)_2_D (4). FGF23-related hypophosphatemic diseases include various genetic conditions, such as X-linked hypophosphatemic rickets (XLH, MIM #307800), autosomal dominant hypophosphatemic rickets (ADHR, MIM #193100), autosomal recessive hypophosphatemic rickets 1 (ARHR1, MIM #241520), ARHR2 (MIM #613312), and Raine syndrome (RNS, MIM #259775) (4).

Osteocytes are dendritic-shaped cells that terminally differentiate from a subpopulation of matrix-producing osteoblasts (5). They reside in lacunae, small cavities surrounded by a mineralized bone matrix, and communicate with other osteocytes and cells on bone surface including osteoblasts, by their long processes in canaliculi. Although research on their function has been hindered because of their location and inaccessibility, scientific advances in the past few decades have revealed that osteocytes play critical roles in the bone homeostasis and the regulation of Pi metabolism (5). The identification and characterization of the human osteocytic genes responsible for rare skeletal diseases have contributed to a more detailed understanding of osteocyte function. *SOST* was identified as the causative gene for sclerosteosis 1 (MIM #269500) and van Buchem disease (MIM #239100), which are characterized by a high bone mass, indicating that osteocytes regulate bone mass by producing sclerostin (6, 7). In addition to *FGF23*, several causative genes for hereditary hypophosphatemic rickets are highly expressed in osteocytes. Among them are the *phosphate-regulating gene homologous to endopeptidase on X chromosome (PHEX)*, *dentin matrix protein 1 (DMP1)*, and *family with sequence similarity 20, member C (FAM20C).* Inactivating mutations in these genes cause hypophosphatemia and impaired skeletal mineralization by increasing the production of FGF23 in osteocytes (8).

This article aims to summarize and discuss the current knowledge on the roles of osteocytes in the pathogenesis of hypophosphatemic rickets.

## Pi homeostasis and FGF23

Pi homeostasis is mainly maintained by the balance between its absorption in the intestines, excretion from the kidneys, and deposition in and mobilization from bone and involves endocrine factors, such as 1,25(OH)_2_D, parathyroid hormone (PTH), and FGF23 (1). 1,25(OH)_2_D increases intestinal Pi absorption by up-regulating the expression of type IIb Na^+^/Pi co-transporter NaPi-IIb (1, 9). PTH increases Pi excretion by reducing the abundance of NaPi-IIa and NaPi-IIc in the brush border membrane of proximal tubules (1, 9). FGF23, whose main source are the osteocytes in bone, down-regulates the renal expression of NaPi-IIa and NaPi-IIc to increase Pi excretion and lower serum Pi levels. FGF23 also decreases the production of 1,25(OH)_2_D, which reduces intestinal Pi absorption and further lowers serum Pi levels (3).

Bioactive intact FGF23 is cleaved to be inactivated between Arg^179^ and Ser^180^ by a subtilisin-like proprotein convertase (10). The *O*-glycosylation of Thr^178^ in FGF23 prevents the cleavage-mediated inactivation of FGF23 (11). Intact FGF23 assays utilize two antibodies against the N- and C-terminal segments of the cleavage site and capture only bioactive intact proteins (12). On the other hand, C-terminal assays use antibodies against the C-terminal segments of the cleavage site, and detect both the intact protein and cleaved C-terminal fragments of FGF23 (13). FGF23 at physiological concentrations requires αKlotho as a cofactor to exert signals through FGF receptors (FGFR) in its distant target organs (14, 15).

## Osteocytes and bone homeostasis

In the adult skeleton, osteocytes represent more than 90% of all bone cells (5). They control bone mass by producing sclerostin encoded by the *SOST* gene. Sclerostin inhibits Wnt/β-catenin signaling by binding to low-density lipoprotein receptor-related protein 5 (LRP5) and LRP6 (16). Wnt/β-catenin signaling plays important roles in bone homeostasis: the activation of this pathway facilitates the commitment of mesenchymal progenitor cells to the osteoblast lineage and promotes the proliferation and differentiation of committed osteoblasts (17, 18). In addition, this pathway suppresses the differentiation and activation of osteoclasts by reducing the expression ratio of receptor activator of nuclear factor κ B ligand (RANKL) to osteoprotegerin in osteoblast lineage cells (17). A sclerostin deficiency due to inactivating variants and a large deletion in the regulatory sequence of the *SOST* gene cause sclerosteosis 1 and van Buchem disease, respectively, which are characterized by osteosclerosis and a high bone mass (6, 7). Mechanical force and PTH suppress the expression of sclerostin/SOST in osteocytes, leading to enhanced Wnt/β-catenin signaling and an increased bone mass (19–22).

Inhibition of sclerostin provides a bone anabolic treatment for bone diseases with a low bone mass. The anti-sclerostin antibody romosozumab is widely used to treat patients with osteoporosis at a high risk of fracture (23). A randomized phase 2a trial on adult patients with moderate osteogenesis imperfecta demonstrated that treatment with the anti-sclerostin antibody BPS804 increased the areal bone mineral density of the lumber spine by stimulating bone formation and reducing bone resorption (24).

RANKL, which plays an essential role in osteoclastogenesis and the activation of osteoclasts, is expressed in osteocytes and osteoblasts. The conditional deletion of RANKL from osteocytes in mice resulted in a progressive osteopetrotic phenotype after birth (25, 26), and studies using several Cre drivers suggested that osteocyte-derived RANKL, rather than osteoblast-derived RANKL, plays a major role in bone remodeling (27). Osteocyte-derived RANKL is up-regulated by aging and may be involved in the aging-related loss of cortical bone (28). Therefore, osteocytes are essential in the regulation of both bone formation and resorption.

## Osteocytes and the pathogenesis of FGF23-related hypophosphatemic rickets

### FGF23-related hypophosphatemic rickets/osteomalacia

Disorders associated with the excessive effects of FGF23 manifest hypophosphatemic rickets/osteomalacia characterized by renal Pi wasting and inappropriately normal or low levels of serum 1,25(OH)_2_D, and are collectively called FGF23-related hypophosphatemic rickets/osteomalacia (4). This includes various conditions such as tumor-induced osteomalacia caused by the overproduction of FGF23 due to phosphaturic mesenchymal tumors, hypophosphatemia associated with the intravenous administration of iron preparations, and genetic disorders, such as ADHR, XLH, ARHR1, ARHR2, and RNS (4).

### ADHR and osteocytes

ADHR is caused by missense mutations at the Arg^176^ or Arg^179^ residues within the RXXR/S motif of FGF23, which is recognized by subtilisin-like proprotein convertase (29, 30). Although these mutations are expected to confer resistance to the cleavage-mediated inactivation of FGF23, ADHR shows incomplete penetrance (31). The delayed onset of ADHR generally manifests in females after puberty, and an iron deficiency has been suggested to drive increases in serum FGF23 levels and manifestation of the disease (32). The iron status modified the plasma levels of intact and C-terminal fragments of FGF23 differently in ADHR patients and healthy subjects (32). In animal studies, a low-iron diet caused hypophosphatemia in ADHR model mice carrying FGF23[p.R179Q] mutation, along with increased serum levels of both intact FGF23 and C-terminal fragments. On the other hand, wild-type mice on a low-iron diet showed normal intact FGF23 levels and normophosphatemia despite elevated C-terminal FGF23 levels (33). *In vitro* experiments using an iron chelator have suggested that an iron deficiency up-regulates the expression of *Fgf23* in osteoblastic cells *via* the activation of hypoxia inducible factor 1α (33). These findings suggest that, in an iron deficiency, the wild-type FGF23 protein is excessively inactivated by proteolytic cleavage to maintain normal serum Pi levels, whereas the impaired cleavage of FGF23 with an ADHR mutation increases bioactive FGF23 levels and promotes hypophosphatemia. Therefore, sensing of the iron status by osteocytes appears to be a critical step for the onset of ADHR.

### XLH and osteocytes

XLH is the most common form of hereditary hypophosphatemic rickets and is caused by inactivating mutations in the *PHEX* gene (34). XLH patients have elevated serum levels of intact FGF23, resulting in renal Pi wasting, hypophosphatemia, and low to inappropriately normal levels of serum 1,25(OH)_2_D (35). Pediatric patients may manifest rickets, bone deformities, and a short stature, while adult patients may show osteomalacia, fractures, pseudo-fractures, enthesopathies (pathological changes in the insertion of tendons, ligaments, and joint capsules), osteoarthritis, hearing loss, and hyperparathyroidism (35). To date, more than 1000 *PHEX* mutations have been identified to be responsible for XLH (https://www.rarediseasegenes.com/).

Similar to *FGF23*, *PHEX* is expressed in osteoblast lineage cells with higher expression in osteocytes (8). Hypomineralized periosteocytic lesions are unique features in the bones of XLH patients, indicating abnormal osteocyte functions (36). Hypophosphatemic *Hyp* mice, which carry a large deletion in the *Phex* gene, reproduce elevated FGF23 levels and hypophosphatemia and are widely used as a murine model for XLH (37, 38). The conditional ablation of the *Phex* gene from osteoblasts and osteocytes using Cre recombinase driven by the *Osteocalcin* promoter resulted in an almost identical phenotype to that of *Hyp* mice, suggesting that impaired PHEX functions in osteoblasts and/or osteocytes alone are sufficient to cause the disease (39).

The PHEX protein is suggested to function as a cell surface-bound, zinc-dependent protease based on its structure (40): however, its precise physiological roles and the mechanisms by which its deficiency increases FGF23 levels remain elusive. Although there is no clear relationship between the *PHEX* genotype and clinical phenotype (41), a recent study using 3-dimensional modeling has suggested that serum FGF23 levels are higher in patients whose *PHEX* mutations affect the zinc-binding site or the cavity of the protein (42). Thus, these regions may be important for PHEX function to regulate FGF23.

Matrix extracellular phosphoglycoprotein (MEPE) belongs to the SIBLING (small integrin-binding ligand, N-linked glycoproteins) family. PHEX binds to MEPE *via* the acidic serine-aspartate rich MEPE-associated motif (ASARM) located at the C-terminal region of MEPE, immediately downstream of cathepsin B recognition site (43). ASARM peptides produced by proteolytic cleavage of MEPE inhibit mineralization (44). Serum levels of ASARM peptides are increased in *Hyp* mice, implicating that they are degraded by PHEX and that their accumulation might be involved in the defective skeletal mineralization in *PHEX* deficiency (45).

The ASARM motif is shared in other members of SIBLINGs family, such as osteopontin (OPN) and DMP1 (46). Synthesized OPN-ASARM peptides inhibited *in vitro* mineralization of osteoblasts by binding to hydroxyapatite, and addition of recombinant PHEX protein rescued this inhibition of mineralization (46). OPN is highly expressed in osteoblasts and osteocytes and can be a candidate of protein substrate for PHEX (47). A recent study demonstrated that the genetic ablation of OPN in *Hyp* mice partially rescued impaired skeletal mineralization without correcting hypophosphatemia (48), suggesting the involvement of OPN in the pathogenesis of XLH.

**Figure 1:**
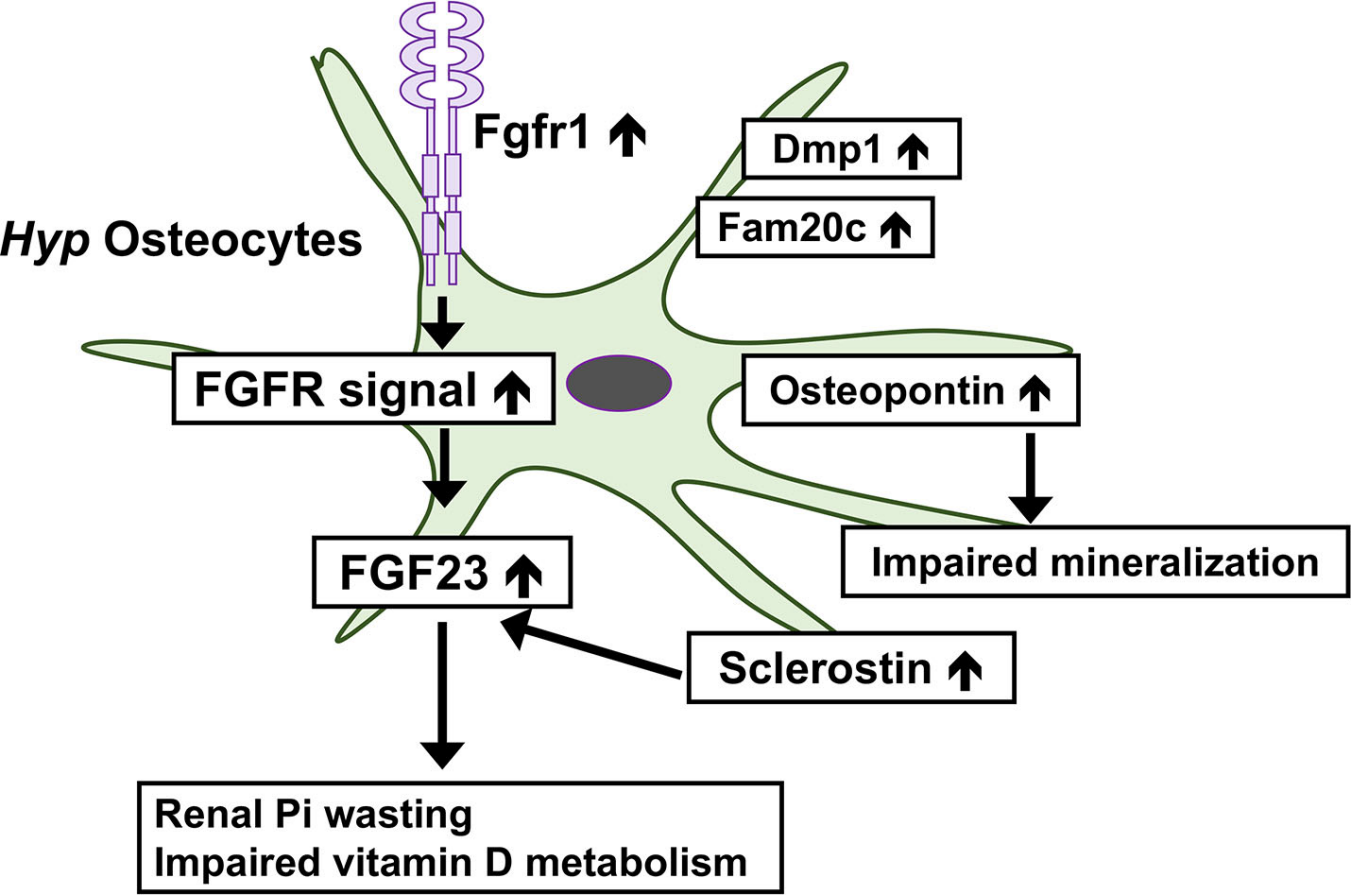
Complex abnormalities in osteocytes of *Phex*-deficient *Hyp* mice. In *Hyp* osteocytes, the expression of *Fgfr1* is increased and enhanced FGFR signaling may underlie the overproduction of FGF23. The overproduction of FGF23 leads to the renal wasting of inorganic phosphate (Pi) and impaired vitamin D metabolism. *Hyp* osteocytes produce an increased amount of sclerostin, which may stimulate the production of FGF23. The production of osteopontin is up-regulated in *Hyp* osteocytes, which impairs skeletal mineralization. The expression of *Dmp1* and *Fam20c* is also increased.

FGFR signaling is enhanced in *Hyp* bone (49). Comparisons of gene expression in osteocytes between *Hyp* mice and wild-type mice revealed marked increases in the osteocytic expression of canonical FGF ligands (*Fgf1* and *Fgf2*), FGF receptors (*Fgfr1-3*), and *early growth response* 1, a downstream target for FGFR activation, in *Hyp* mice (8). Furthermore, the osteocyte-specific deletion of *Fgfr1* partially restored the overproduction of FGF23 and ameliorated hypophosphatemia and rickets in *Hyp* mice (50). These findings suggest a pathogenic role for enhanced FGFR signaling in the overproduction of FGF23 in *Phex*-deficient osteocytes. This is of interest because previous studies suggested the involvement of FGFR in Pi sensing (1). In various cell types including osteoblasts, a treatment with high Pi activates FGFR to regulate gene expression (51–53). In the osteoblastic cell line MC3T3-E1, a treatment with an FGFR inhibitor abolished the up-regulation of *Dmp1* by increasing extracellular Pi levels (53). In mice, the Pi-induced activation of FGFR1 up-regulated the *Galnt3* expression in bone, resulting in an elevation of serum FGF23 levels (54). Enhanced FGFR signaling in *Hyp* osteocytes suggests that dysregulated Pi sensing might underlie the pathogenesis of XLH. In *Hyp* osteocytes, the expression of *Dmp1* and *Fam20c* was also markedly up-regulated, indicating complex abnormalities in *Phex*-deficient osteocytes (8). **Figure 1** shows complex abnormalities in *Hyp* osteocytes.

### ARHR1 and osteocytes

ARHR1 is caused by inactivating mutations in the *DMP1* gene, which encodes an extracellular matrix protein belonging to the SIBLINGs family (55, 56). *DMP1* is predominantly expressed in osteocytes and odontoblasts. *Dmp1*-null mice show phenotypes reproducing the clinical manifestation in ARHR1 and exhibit defective osteocyte maturation, increased FGF23 expression in osteocytes, and impaired skeletal mineralization (56). Similar to *Phex*-deficient *Hyp* mice, the activation of the FGFR pathway was suggested to be involved in the increased production of FGF23 in *Dmp1*-null mice (49). The overproduction of FGF23 may be attributed to enhanced FGFR signaling in osteocytes in both XLH and ARHR1.

It was reported that CRISPR/Cas9-mediated ablation of *DMP1* in rabbit caused the phenotype similar to human ARHR1, including the elevated serum FGF23 and hypophosphatemic rickets. *DMP1*-knockout rabbits also exhibited severe defects in bone microarchitecture (57).

### ARHR2 and osteocytes

ARHR2 is caused by inactivating mutations in the *ectonucleotide pyrophosphatase phosphodiesterase-1* (*ENPP1*) gene, which encodes an ectoenzyme that catalyzes the hydrolysis of ATP to AMP and inorganic pyrophosphate (PPi) (58–60). PPi inhibits mineralization, and inactivating mutations in *ENPP1* cause disorders characterized by ectopic calcification, such as generalized arterial calcification of infancy (60, 61). *ENPP1* is expressed in many tissues, and is highly expressed in chondrocytes, osteoblasts, and vascular smooth muscle cells (62). The mechanisms by which an ENPP1 deficiency leads to the overproduction of FGF23 currently remain elusive. PPi may regulate the FGF23 production: however, patients with hypophosphatasia, the disease caused by inactivating mutations in tissue non-specific alkaline phosphatase, have normal FGF23 levels along with elevated PPi levels (63). A recent mouse study demonstrated the increased expression of *Fgf23* in the bones of *Enpp1*-deficient mice, and a negative correlation between *Enpp1* and *Fgf23* transcription was revealed by dosing *Enpp1*-deficient mice with ENPP1-Fc recombinant protein (64). These findings suggest that the lack of osteocyte-derived Enpp1 plays a role in the pathogenesis of ARHR2.

### RNS and osteocytes

RNS is a disease of autosomal recessive inheritance, and the patients show craniofacial malformation, osteosclerotic bone dysplasia, and a poor prognosis. RNS patients who survive infancy may manifest hypophosphatemia related to FGF23 excess and dental abnormalities (65, 66). The gene responsible for RNS is *FAM20C* (also called *DMP4*), which encodes a kinase that phosphorylates various secreted proteins including FGF23 and members of the SIBLING family, such as DMP1 (10, 67, 68). The expression of *Fam20c* was higher in osteocytes than in osteoblasts, which was similar to the expression pattern of *Fgf23*, *Dmp1* and *Phex* (8). Wang, et al. generated *Fam20c* conditional knockout mice, in which the gene was globally deleted or specifically inactivated in the mineralized tissues (69). Both mouse lines exhibited hypophosphatemic rickets along with elevated FGF23 levels in serum and bone. They also demonstrated the down-regulation of a number of marker genes in osteoblast/osteocyte lineage cells, which included *Dmp1* and *osteocalcin* (69). Furthermore, knockout of *Fam20c* in immortalized murine osteoblasts resulted in the up-regulation of *Fgf23* and down-regulation of *Dmp1* (70). Thus, Fam20c-deficiency may lead to the overproduction of FGF23 in bone through thedown-regulation of the *Dmp1* expression and impaired phosphorylation of Dmp1 protein. However, a transgenic overexpression of *Dmp1* failed to rescue elevated FGF23 levels and hypophosphatemia of *Fam20c* knockout mice (71), suggesting the involvement of other mechanisms. Since it was reported that FAM20C phosphorylated FGF23 on Ser^180^, which prevented the *O*-glycosylation of FGF23 on Thr^178^ and accelerated cleavage-mediated inactivation (10),the impaired cleavage of FGF23 due to the reduced phosphorylation of FGF23 on Ser^180^ may be one of the pathogenesis of elevated intact FGF23 in RNS.

## Osteocytes as the target to treat FGF23-related hypophosphatemic rickets

As conventional medical therapy for FGF23-related hypophosphatemic rickets/osteomalacia, oral Pi salts and active vitamin D analogues have been administered to correct hypophosphatemia and reduced levels of 1,25(OH)_2_D (35). A previous study reported that the initiation of conventional therapy in early infancy improved the growth and biochemical and radiological outcomes of pediatric patients with XLH; however, it failed to completely normalize skeletal development (72). Furthermore, conventional therapy did not prevent or attenuate enthesopathies or hearing loss in adult patients (35). Moreover, the administration of oral Pi salts and active vitamin D may cause adverse effects, such as hypercalciuria, nephrocalcinosis, and secondary/tertiary hyperparathyroidism (35).

Burosumab, a humanized anti-FGF23 monoclonal antibody, has been approved since 2018 for the treatment of XLH in several countries based on the promising findings of the clinical trials (73–76). The approved indication for burosumab differs among regions/countries; it is approved for patients with TIO in several countries. In Japan, it is approved for all patients with FGF23-related hypophosphatemic rickets/osteomalacia (4).

Since FGF23 and sclerostin are both expressed in osteocytes, it is reasonable to hypothesize that they may be related. Serum sclerostin levels were reported to be higher in XLH patients than in healthy controls (77, 78). An *in vitro* study suggested that sclerostin directly stimulated the production of FGF23 (79). The treatment of *Hyp* mice with an anti-sclerostin antibody suppressed serum intact FGF23 levels and increased serum Pi levels and bone mass (80, 81), suggesting the potential of inhibiting sclerostin as a treatment option for XLH.

## Conclusion

In addition to their profound roles in bone homeostasis, osteocytes embedded in the bone matrix play a central role in Pi metabolism as the main source of FGF23. Osteocytes also express PHEX, DMP1, ENPP1, and FAM20C, and inactivating mutations cause FGF23-related hypophosphatemic rickets/osteomalacia. Although the mechanisms by which inactivating mutations in these genes cause the overproduction of FGF23 have not yet been clarified, enhanced FGFR signaling and abnormal Pi sensing might be involved in the pathogenesis of XLH and ARHR1. Serum sclerostin levels are elevated in XLH patients, indicating complex abnormalities in osteocytes. Further studies are needed to elucidate the precise roles of osteocytes in the pathogenesis of hereditary hypophosphatemic diseases, which will contribute to the development of better treatments for these conditions.

## Author contributions

MY and TM developed the concept and prepared the manuscript. All authors contributed to the article and approved the submitted version.

## Funding

Preparation of the manuscript was supported by a grant from Japan Society for the Promotion of Science (JSPS KAKENHI Grant Number 21K07835) to TM.

## Conflict of interest

The authors declare that the research was conducted in the absence of any commercial or financial relationships that could be construed as a potential conflict of interest.

## Publisher’s note

All claims expressed in this article are solely those of the authors and do not necessarily represent those of their affiliated organizations, or those of the publisher, the editors and the reviewers. Any product that may be evaluated in this article, or claim that may be made by its manufacturer, is not guaranteed or endorsed by the publisher.
